# An analysis of teachers’ instructions and feedback at a contemporary dance university

**DOI:** 10.3389/fpsyg.2023.1133737

**Published:** 2023-04-27

**Authors:** Benjamin F. Soerel, Larissa A. Plaatsman, Jolan Kegelaers, Janine H. Stubbe, Rogier M. van Rijn, Raôul R. D. Oudejans

**Affiliations:** ^1^Codarts University of the Arts, Rotterdam, Netherlands; ^2^Breederode Hogeschool, Rotterdam, Netherlands; ^3^Department of Human Movement Sciences, Amsterdam Movement Sciences, Vrije Universiteit Amsterdam, Amsterdam, Netherlands; ^4^Performing Artist and Athlete Research Lab, Codarts University of the Arts, Rotterdam, Netherlands; ^5^Faculty of Psychology and Educational Sciences, Vrije Universiteit Brussel, Brussels, Belgium; ^6^Faculty of Physical Education and Physiotherapy, Vrije Universiteit Brussel, Brussels, Belgium; ^7^Department of General Practice, Erasmus MC University Medical Centre Rotterdam, Rotterdam, Netherlands; ^8^Rotterdam Arts and Sciences Lab, Rotterdam, Netherlands; ^9^Institute for Brain and Behavior, Amsterdam, Netherlands; ^10^Faculty of Sports and Nutrition, Amsterdam University of Applied Sciences, Amsterdam, Netherlands

**Keywords:** coaching, instructions, feedback, focus of attention, modern dance, observation

## Abstract

**Background:**

Given the demands posed by excessive practice quantities in modern dance, physical and mental health can be compromised. Therefore, there is a need to consider how quality of practice may be improved and possibly even reduce training times. Sports literature has shown that instructions and feedback given by coaches can have an effect on the quality of training and influence self-regulation and the performance of athletes. However, currently little is known about the use of instructions and feedback by dance teachers. The aim of the current study was, therefore, to examine the type of instructions and feedback given by dance teachers during various dance classes.

**Methods:**

A total of six dance teachers participated in this study. Video and audio recordings were made of six dance classes and two rehearsals at a contemporary dance university. The dance teacher’s coaching behavior was analyzed using the modified Coach Analysis and Intervention System (CAIS). Additionally, feedback and instructions were also examined in terms of their corresponding focus of attention. Absolute numbers, as well as times per minute (TPM) rates were calculated for each behavior before, during, and after an exercise. Absolute numbers were also used to calculate ratios of positive-negative feedback and open-closed questions.

**Results:**

Most feedback comments were given after an exercise (472 out of 986 total observed behaviors). Improvisation had the highest positive-negative feedback ratio (29) and open-closed questions ratio (1.56). Out of the focus of attention comments, internal focus of attention comments were used most frequently (572 out of 900).

**Discussion/conclusion:**

The results make clear that there is a large variability in instructions and feedback over teachers and classes. Overall, there is room for improvement toward a higher positive-negative feedback ratio, a higher open-closed question ratio and producing more comments eliciting an external focus of attention.

## Introduction

Pre-professional ballet and modern dancers, including vocational dance students, typically face a large number of training hours per week (Kenny et al., 2016; Jeffries et al., 2017; Stubbe et al., 2022). Insufficient rest relative to the number of training hours increases the risk of injury and could, in combination with other factors, lead to burnout in dancers (Koutedakis, 2000; Twitchett et al., 2010). In turn, both physical and mental health issues often lead to a reduction in training volume and subsequent performance (van Winden et al., 2020). Given the demands posed by excessive practice quantities in dance, there is a need to consider how quality of practice may be improved. By optimizing the quality of dance classes, it may be possible to achieve more in less training time, which in turn could have positive consequences for dancers’ physical and mental health (Koutedakis, 2000; Twitchett et al., 2010). Crucially, advances in providing optimal coaching behaviors from fields such as performance psychology or skill acquisition, often developed in other domains (e.g., sport, music), could provide key insights into how practice may be improved, but have only sporadically found their way into dance teaching.

One particular way the quality of training in dance practice may be improved, relates to examining how information is transferred; or in other words, considering dance teachers’ use of instructions and feedback (Ericsson et al., 1993; Williams and Hodges, 2005; Neenan, 2009; Badami et al., 2012; Abbas and North, 2018; Otte et al., 2020; Larkin et al., 2022). In dance, instructions provide the information about the execution of the skills whereas feedback functions as confirmation, motivation, and guidance for the correction of mistakes (Wulf et al., 1998; Enghauser, 2003; Gibbons, 2004). Instructions and feedback aim to direct the attention of performers to relevant salient information for task execution and further development of expertise. Hence, the use of adequate instructions and feedback has a crucial role in supporting, guiding, and complementing the learning process (Otte et al., 2020). This makes verbal and non-verbal information by dance teachers an important qualitative component of training and deliberate practice (Ericsson et al., 1993). In the following section, we will briefly discuss several features of dance teachers’ instructions and feedback which may have an impact on students’ learning and performance.

### Positive versus negative feedback

The instructions and feedback provided by teachers or coaches can vary in terms of its emotional valence (Otte et al., 2020). For example, praising, motivating and constructive feedback, can be considered positive and supportive; whereas criticisms, scolding, or disparagement can be considered negative feedback. Research suggests that giving higher rates of positive feedback, as opposed to negative feedback, can lead to a more effective learning, improved performance, as well as higher intrinsic motivation and confidence in one’s abilities (Badami et al., 2012; Abbas and North, 2018). Strengthening self-confidence can, in turn, lead to the automaticity, effortlessness and a task focus seen in effective high-level performance (Wulf and Lewthwaite, 2016). Therefore, it may be advised that dance teachers prioritize supportive and positive feedback, whilst limiting negative feedback (Otte et al., 2020).

### Direct instruction versus questioning

Instructions and feedback from coaches can also be distinguished in terms of their aim to provide information in a direct prescriptive manner or in a questioning form to elicit reflection and self-awareness from the student (Otte et al., 2020). Research from sports suggests that overuse of direct verbal instructions and feedback may actually impede learning and development by hindering athletes’ self-exploratory and self-regulating mechanisms (Davids et al., 2008; Partington and Cushion, 2013). In order to counteract the dependence on direct instructions and feedback, the use of questions has been proposed as a way to strengthen learners’ self-regulation (Williams and Hodges, 2005; Elferink-Gemser and Hettinga, 2017; Otte et al., 2020). Questions can further be distinguished in terms of their open or closed nature. Open questions typically offer an unlimited number of answer possibilities and directly elicit cognitive engagement from the learner. In contrast, the formulation of closed question typically allows a limited number of answer-options (Raya-Castellano et al., 2020). Asking an open question (e.g., “What is the best way to stay on balance?”) as opposed to a closed question (e.g., “Were your hips placed above your feet when you tried to balance?”) could stimulate self-reflection in a student and can subsequently contribute to self-regulation (Neenan, 2009; Elferink-Gemser and Hettinga, 2017). Furthermore, asking open questions can increase a dancers’ sense of agency or autonomy, positively affecting the ability to acquire a skill and improve performance (Wulf and Lewthwaite, 2016).

### Focus of attention

Different instructions and feedback can equally vary in terms of the attentional focus they elicit in the learner (Wulf et al., 1998; Wulf, 2013). The attentional focus elicited by the information provided by a teacher can be either internally focused or externally focused (Wulf, 2013). An internal focus refers to directing one’s attention to specific body movements. A simple example in dance could be “stretch your arm as far as possible.” An external focus on the other hand refers to directing one’s attention to the intended effect of a movement, rather than the movement itself. An alternative instruction to the previous example, this time eliciting an external focus of attention, could be something like: “stretch as if trying to touch the wall.” Out of these two types of focus of attention, an external focus of attention has consistently been found to be most effective and efficient for motor learning and performance across multiple domains, as illustrated by improved results for balance, accuracy in hitting a target, precision in producing a demanded amount of force, and reduced muscle activity, heart rate, and oxygen uptake when performing a motor task (Wulf, 2013; Wulf and Lewthwaite, 2016). Similarly, the use of metaphors, where an external focus of attention is elicited, could have similar effects on motor learning and performance (Wulf and Lewthwaite, 2016; Otte et al., 2020). In dance, one study examining the use of internal and external focus of attention found that the majority of professional ballet dancers tended to use either an internal focus alone or a combination of both attentional foci (Guss-West and Wulf, 2016).

### Instructions and feedback in dance

As briefly summarized above, research from across different performance domains, has shown that components of teachers’ instructions and feedback can have differential effects on learning and performing motor tasks (Williams and Hodges, 2005; Neenan, 2009; Badami et al., 2012; Abbas and North, 2018; Otte et al., 2020; Larkin et al., 2022). However, relatively little is known about teachers’ use of instructions and feedback in dance specifically. Moreover, the limited research that is available has primarily relied on the use of retrospective interviews or questionnaires to evaluate instructions and feedback given by teachers (Van Rossum, 2004; Rafferty and Wyon, 2006; Klockare et al., 2011). For example, Klockare and colleagues used semi-structured interviews to examine dance teachers’ feedback and instructions, specifically aimed toward developing key psychological skills. The authors found that teachers emphasized the importance of positive feedback, whilst at the same time finding it difficult to provide such positive feedback and more commonly resorting to providing criticisms and corrections (Klockare et al., 2011). Both Van Rossum (2004) and Rafferty and Wyon (2006) used an adapted version of the Leaderschip Scale for Sport (LSS) questionnaire to compare dance teachers’ and students’ perceptions of optimal feedback and instruction. Both studies found that teachers and students alike identified “feedback” and “Training and instruction” as the two most desirable dimensions of an ideal teacher. However, interesting discrepancies were also found between the perceptions of teachers and students, indicating that the students perceived teachers to give less positive feedback and use more autocratic behavior compared to the teachers themselves (Van Rossum, 2004; Rafferty and Wyon, 2006).

These studies provide initial insight into how dance teachers and students experience coaching behavior, instructions and feedback in dance training. However, these findings are limited in that they are based on the perceptions of teacher and students, rather than observations of actual coaching behaviors. Therefore, observation research studying teachers’ behavior *in situ* during actual dance classes can provide an important advancement of the current literature. Thus, the aim of the current study was, thus, to quantitatively determine the coaching behaviors of dance teachers during various dance classes and rehearsals and to gain insight into the way teachers provide instructions and feedback. Such knowledge may provide a small first step in helping to understand how dance teachers in the future can optimize their practice designs.

## Materials and methods

### Participants

The current study used an observational design to examine the instructions and feedback of dance teachers during dance classes and rehearsals provided to a minimum of 12 and a maximum of 27 first-year Bachelor contemporary dance students at Codarts University of the Arts (Rotterdam, The Netherlands). A total of six dance teachers, three men (*M*_age_ = 59.33; SD = 4.93) and three women (*M*_age_ = 57; SD = 14.53), participated in this study. The six teachers were followed during six dance classes and two rehearsals. The dance classes included a Modern Jazz class, an Improvisation class, two Ballet classes and two Contemporary Dance classes, namely one class of Graham and one of Laban. Both rehearsals were Graham repertoire, done in preparation of an upcoming performance. Modern Jazz is a dance style influenced by African American jazz dance and Caribbean traditional dance, which has developed into a theater-based performance form of dance. During Improvisation classes dancers are presented with an assignment and have to come up with their own movement vocabulary. Ballet is a traditional dance style with strict technical rules and aims for optimal aesthetics, such as making long lines and high extensions. Contemporary Dance styles are expressionistic dance styles. Martha Graham and Rudolf von Laban were two important choreographers from the beginning of the 20th century, who created their own dance technique during the uprise of the Contemporary Dance movement. Ballet classes were given by two different teachers and both Graham and the rehearsals were given by the same teacher. In the dance world, Codarts University of the Arts is seen as a representative and well-known contemporary dance university.

### Material

#### Cameras

Video recordings were made with three GoPro cameras (type; HERO 3 and 4). Two cameras (labeled *overview cameras*) were placed at opposite corners of the dance studio at a minimum height of two meters to create an overview of the whole studio. A third GoPro camera (referred to as *feedback camera*) was attached to a chest harness worn by the teachers. This camera was primarily used to examine the vantage point of the teacher and record verbal communication offered by the teacher.

#### Assessment coach behavior

The coaching behavior (i.e., instructions and feedback) of the dance teachers was assessed using an adapted version of the Coach Analysis and Intervention System (CAIS; Cushion et al., 2012). The CAIS was originally developed for use in sport and is a valid analysis system to identify specific behaviors that can occur in a coaching environment (Cushion et al., 2012; Partington and Cushion, 2013). The CAIS is scored based on video recordings made during training sessions and/or sports competitions, or in our case dance classes and rehearsals. The system includes 23 primary behaviors related to physical behavior, instruction, feedback/reinforcement, (non-)verbal behavior, questioning and management. It is not uncommon to adapt the CAIS to a study’s context and research question (Harvey et al., 2013; Partington et al., 2014). For the present study, some items from the original CAIS were removed, added or merged. This was done in function of the specific research question formulated in this study and based primarily upon the expertise of the first and third author, who both have extensive applied experience as a high-performance dancer. For example, the original CAIS included three forms of management (i.e., direct, indirect, criticism) (Cushion et al., 2012). As management behaviors were not the focus of the current study, the choice was made to collapse these behaviors into a single category. Moreover, the use of cues was not included in the original CAIS. However, both verbal and non-verbal (e.g., snapping fingers, tapping feet) cues are very commonly used techniques within dance instruction (Enghauser, 2003). As such, a cue category was added to the CAIS for the current study. All included 16 behaviors, including adopted abbreviations, used for the current study are presented in Table 1.

**TABLE 1:** Overview of the adapted coach analysis and intervention system.

Behaviors	Explanation
Management (man)	Arranging matters that may or may not be related to the class, such as organizing the music and organizing groups. e.g., “I need you in two groups of eight.”
Instructions (ins)	Information to instruct/direct skill or movement of the dancers’ performance. e.g., “Lift your leg to a la seconde,” “Pull your shoulder blades down,” “We do tendu, 2, 3, 4, and close, 6, 7, 8.”
Corrective feedback (cor)	Corrective statements that contain information that *specifically aim to improve the dancer(s) performance at the next skill attempt* (can be delivered concurrently or post). e.g., “It would help if you step out further” and ‘”probably do not want to force your turn out.”
Cues (cue)	Brief reminders about previously given information. e.g., “Point,” “hold,” and “step out”
Open question (open)	A question with a large number of answer options and which can be of long duration. e.g., “What is the best way to stay on balance?”
Closed question (closed)	A question with a limited number of possible answers and such as a yes/no question. e.g., “Were your hips placed above your feet when you tried to balance?”
General positive feedback (gpf)	General positive or supportive verbal statements OR non-verbal gestures (can be delivered concurrently or post). e.g., “Well tried,” “well done,” “much better,” “lovely,” applauding, and thumbs up.
General negative feedback (gnf)	General negative or unsupportive verbal statements OR non-verbal gestures (can be delivered concurrently or post). e.g., “Don’t do that again,” “that was rubbish,” shaking head, and thumbs down.
Specific positive feedback (spf)	Specific positive or supportive verbal statements *that specifically aim to provide information about the quality of performance* (can be delivered concurrently or post). e.g., “Good turn” and “nice balance.”
Specific negative feedback (snf)	Specific negative or unsupportive verbal statements *that specifically aim to provide information about the quality of performance* (can be delivered concurrently or post). e.g., “Your turn is too slow” and “you are not dancing on the music.”
Positive modeling (pos)	Skill-demonstration- with or without verbal instruction that shows performer the correct way to perform.
Negative modeling (neg)	Skill-demonstration- with or without verbal instruction that shows performer the incorrect way to perform.
Physical assistance (pa)	Physically moving the performer’s body to the proper position or through the correct range of movement.
Humor (hum)	Jokes or content designed to make dancers laugh or smile. e.g., “Have you been eating jumping beans for breakfast?”
Answer to question (ans)	The teacher’s answers to a student’s question.
Hustle (hus)	Verbal statements or gestures *linked to effort* to activate or intensify previously directed behavior. e.g., “You can do it,” “come on,” and “go, go, go.”

Importantly, the CAIS does not include a distinction between instruction and feedback aimed at eliciting an internal or external focus of attention. Therefore, in addition to using the CAIS, all of the teachers’ comments were also classified as either inducing an internal or external focus of attention. A similar approach has been used in prior observational research examining the use of focus of attention by baseball coaches (van der Graaff et al., 2018). In addition, comments could also be classified as a metaphor if they did not directly refer to a body movement or outcome of a body movement, but rather relied on analogies or metaphors to convey information. An overview and examples of this adopted classification is provided in Table 2.

**TABLE 2:** Classification of focus of attention comments.

Behaviors	Explanation
Internal (int)	The feedback and instructions are given so that they direct focus of attention to how the movements should be performed, referring to specific body parts. e.g., “Turn your head as quickly as possible when attempting a pirouette.”
External (ext)	The feedback and instructions are given so that they direct focus of attention to the effect of the movement in the environment rather than the actual execution of the movement. The different parts of the studio, such as the floor or the ceiling, may be applicable here. e.g., “Keep looking at the red light as long as possible when attempting a pirouette.”
Metaphor (meta)	The feedback and instructions are given through imagery in the form of a metaphor or analogy. (Corrections to) movements can be indicated by means of remarks that are not necessarily dance-related. e.g., “Perform your plié as if there is chewing gum between your knees.”

### Procedure

**FIGURE 1:**
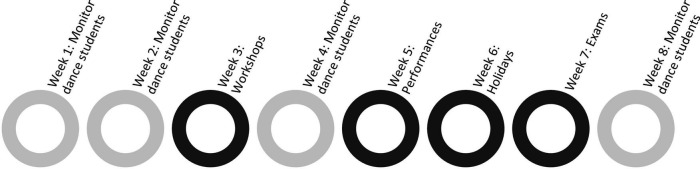
Time line of monitoring the dance students. The gray circles represent the data collection weeks.

Institutions ethics approval (VCWE-2018-142) was obtained prior to the study. Prior to the start of data collection, dance teachers as well as the students were informed about the aims and design of the study. All teachers and students provided written informed consent prior to the study. In agreement with the staff of the dance department, a total of 4 weeks’ (Monday till Friday) worth of videos was collected during dance classes and rehearsals over an 8 weeks period (Figure 1).

These 4 weeks were considered representative training weeks; that is, weeks without unique assignments or deviating practice loads. In agreement with each participating teacher, a standard class and two rehearsals were selected to be filmed with the additional feedback camera. All recorded practice sessions and rehearsals were part of a subset of the classes investigated during the study of Stubbe et al. (2022) assessing objective and subjective training load measures during dance classes. However, the information presented in the current article includes both data and analyses not presented in the original article (Stubbe et al., 2022).

Prior to each class/rehearsal, three GoPro cameras were installed, and recordings were started. When the class or rehearsal was finished, recordings were stopped, and the feedback camera was handed in by the teacher. Collected audiovisual data were then analyzed by the first author, who has extensive experience as a professional dancer. Each recorded class/rehearsal was viewed four times for analysis. The first three viewings used the feedback camera viewpoint. During the first viewing, recordings were used to determine the class structure. This was done by distinguishing times *before*, *during*, and *after* each exercise during each class or rehearsal. The *before* part started the moment the teacher started talking about the new exercise. The *during* part started the moment the music started to play. The *after* part started the moment the last note of the music ended or the final movement of an exercise finished in case the music had already stopped. The end of each exercise part was 1 s before the start of the next part. The second viewing was then used to assess teachers’ behavior before, during and after each exercise using the adapted CAIS. The third viewing was used to assess focus on attention elicited by the teachers’ comments, using the internal, external, or metaphor classification. For this analysis no distinction was made before, during and after an exercise. The final viewing used the overview camera viewpoint to assess whether any non-verbal behaviors were missed that were not visible on the feedback camera. Such non-verbal behaviors could include positive and negative modeling, non-verbal cues, non-verbal forms of management, and non-verbal manifestations of hustle. These behaviors are part of the modified CAIS and were therefore also counted before, during or after each exercise. To keep track of the number of coaching behaviors, a simple tally scoring system was used.

### Analysis

The class and rehearsal structures were determined by calculating the duration *before*, *during* and *after* each exercise. These times were added up for each class and rehearsal to arrive at a total time spent before, during and after exercises. The sum of these times represented the total duration of each class and rehearsal. This data provided a frame of reference to analyze the coaching behavior.

Behaviors from the modified CAIS, scored during the second and fourth viewing, were pooled for each class/rehearsal, resulting in a total number of behaviors before, during and after exercises. Subsequently, for each behavior, the absolute number of behaviors that occurred in period were added up resulting in the total number of behaviors per class/rehearsal. The number of behaviors for internal focus, external focus and metaphor were also calculated as absolute numbers per class/rehearsal and percentages. To calculate the total positive and negative feedback, the absolute numbers of the general and specific feedback were combined. Furthermore, the total amount of feedback delivered in a class/rehearsal was determined by adding the absolute numbers for the general feedback, specific feedback and the corrective feedback. In addition to absolute numbers and percentages, times per minute (TPM) scores were also calculated by dividing the absolute number of behaviors before, during, and after an exercise by the duration of each period in minutes. These TPM are the mean number of comments made per minute and allow for direct comparisons between classes. Means and standard deviations were also calculated for all behaviors.

To determine the interrater reliability of our scoring method, the two first classes were scored independently by two researchers (1st and 2nd author) who both had an extensive background in dance. Interrater reliability was assessed using the agreement percentage for each of the behaviors. A minimum of 80% agreement was considered appropriate for the adapted CAIS and focus of attention assessments to be considered reliable tools to assess the behavior of dance teachers (McHugh, 2012).

## Results

### Interrater reliability

Two classes with a total of 29 exercises were used to calculate the interrater reliabilities. The duration of the before, during and after parts of exercises had a percent agreement of 93%. For the modified CAIS, a total of 843 behaviors were counted by grader 1 and a total of 876 behaviors were counted by grader 2. The total interrater reliability calculated per exercise, per exercise part, per behavior was 87%. For the foci of attention comments a total of 92 focus of attention comments were counted by grader 1 and a total of 99 focus of attention comments were counted by grader 2. The interrater reliability calculated per type of focus of attention was 92%. In all, it can be concluded that the adapted CAIS and focus of attentions assessments are considered reliable tools that can be used for the current data set. Further analyses in this study were, therefore, done using the data of grader 1 only.

### Class duration

**FIGURE 2:**
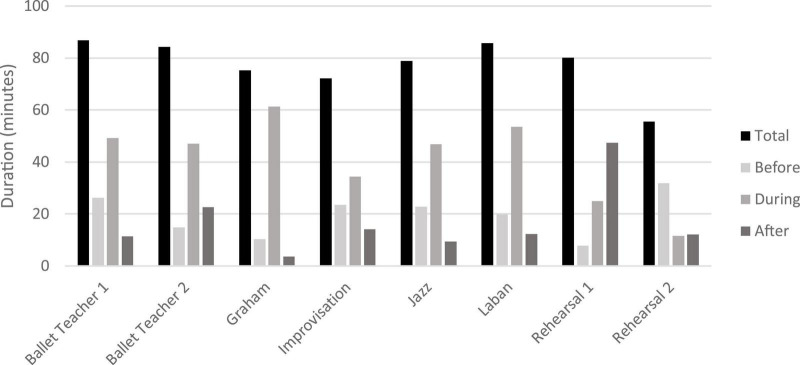
Total class/rehearsal durations.

The total class/rehearsal duration ranged between 72 min and 86 min (*M* = 77.35 min; SD = 10.21). The duration of the parts before (*M* = 19.65 min; SD = 8.18 min), during (*M* = 41.09 min; SD = 16.38 min) and after (*M* = 16.62 min; SD = 13.49 min) an exercise, as well as the total class/rehearsal durations are presented in Figure 2.

### CAIS

Key scores from the CAIS are summarized in Table 3. All CAIS scores can be found as Supplementary appendix.

**TABLE 3:** Absolute numbers (TPM between brackets) for direct feedback comments and questions per class/rehearsal.

	Ballet 1	Ballet 2	Graham	Improvisation	Jazz	Laban	Rehearsal 1	Rehearsal 2
Positive feedback	11 (0.13)	54 (0.64)	82 (1.09)	58 (0.80)	100 (1.27)	64 (0.75)	69 (0.86)	23 (0.41)
Negative feedback	16 (0.18)	46 (0.55)	8 (0.11)	2 (0.03)	6 (0.08)	8 (0.09)	37 (0.46)	21 (0.38)
Ratio pos/neg feedback	0.69	1.17	10.25	29	16.67	8	1.86	1.10
Feedback before exercise	4 (0.15)	2 (0.14)	0 (0)	27 (1.15)	14 (0.62)	12 (0.60)	0 (0)	20 (0.63)
Feedback during exercise	8 (0.16)	85 (1.81)	102 (1.66)	14 (0.40)	87 (1.86)	106 (1.98)	16 (0,64)	17 (1.48)
Feedback after exercise	40 (3.52)	94 (4.17)	23 (6.27)	20 (1.41)	37 (3.96)	49 (3.97)	160 (3.38)	49 (4.0)
Feedback total	52 (0.60)	181 (2.15)	125 (1.66)	61 (0.85)	138 (1.75)	167 (1.95)	176 (2.20)	86 (1.55)
Open questions	2 (0.02)	6 (0.07)	2 (0.03)	59 (0.82)	2 (0.03)	9 (0.10)	2 (0.02)	1 (0.02)
Closed questions	16 (0.18)	37 (0.44)	92 (1.22)	38(0.53)	30 (0.38)	37 (0.43)	78 (0.97)	88 (1.59)
Questions total	18 (0.21)	43 (0.51)	94 (1.25)	97 (1.35)	32 (0.41)	46 (0.54)	80 (1.00)	89 (1.60)
Ratio feedback-questions	2.89	4.21	1.33	0.63	4.31	3.63	2.20	0.97
Ratio open- closed questions	0.13	0.16	0.02	1.56	0.07	0.24	0.03	0.01

### Positive and negative feedback

The positive-negative feedback ratios for all classes/rehearsals ranged between 0.69 and 29 (*M* = 18.5; SD = 14.85). Except for Ballet class 1, all classes/rehearsals had a positive-negative feedback ratio higher than one, indicating more use of positive than negative feedback, except for Ballet class 1. Improvisation had the highest positive-negative feedback ratio (29). Overall, there was a large variety in the total number of positive feedback statements observed, with Jazz having the highest absolute number (100) and TPM (1.27), whereas Ballet 1 had the lowest absolute number (11) and TPM (0.13) and Ballet 2 had the third lowest absolute number (54) and TPM (0.64).

## Direct feedback

TPM of direct feedback ranged between 0.60 for Ballet 1 and 2.20 for Rehearsal 1 (*M* = 1.59; SD = 0.58). With a total of 472 comments (*M* = 59; SD = 46.73) accounting for 47.87% of the direct feedback comments, most feedback was given after an exercise. In contrast, 435 direct feedback comments were observed during exercises (*M* = 54.38; SD = 44.05) accounting for 44.12% of the direct feedback comments and 79 comments before exercises (*M* = 9.88; SD = 10.06) accounting for 8.01% of the direct feedback comments. TPM of direct feedback was highest after exercises for all classes and rehearsals.

### Questions

TPM of questions ranged between 0.21 for Ballet 1 and 1.60 for Rehearsal 2 (*M* = 0.86; SD = 0.51). The absolute number (59) and TPM (0.82) of open questions was highest for Improvisation. The absolute number (92) and TPM (1.22) of closed questions was highest for Graham. All classes/rehearsals had an open-closed question ratio below 1, indicating greater use of closed questions, except for Improvisation. Comparing the rate to which questions were asked compared to the number of direct feedback comments, Improvisation was the only class with a feedback-question ratio below one (0.63).

### Focus of attention

A total of 900 focus of attention comments were observed. These are summarized per class/rehearsal in Table 4. With a total of 572 comments (*M* = 71.5; SD = 72.72) accounting for 63.56% of the total focus of attention comments, internal focus of attention was used the most in all classes/rehearsals, except for Improvisation and Jazz. Metaphor comments were the second most used focus of attention with a total of 183 comments (*M* = 22.88; SD = 21.95) accounting for 20.33% of the total focus of attention comments and external focus of attention comments were used the least with a total of 145 comments (*M* = 18.13; SD = 26.32) accounting for 16.11% of the total focus of attention comments. Graham had the highest absolute number (219) and TPM (2.91) of internal focus comments. Jazz had the highest absolute number (57) and TPM (0.72) of metaphors used. Improvisation had the highest absolute number (82) and TPM (1.14) of external focus comments.

**TABLE 4:** Absolute numbers (TPM between brackets) focus of attention comments per class/rehearsal.

	Ballet 1	Ballet 2	Graham	Improvisation	Jazz	Laban	Rehearsal 1	Rehearsal 2
Internal	80 (0.92)	138 (1.64)	219 (2.91)	16 (0.22)	31 (0.39)	50 (0.58)	25 (0.31)	13 (0.23)
External	9 (0.10)	15 (0.18)	17 (0.23)	82 (1.14)	9 (0.11)	8 (0.09)	3 (0.04)	2 (0.04)
Metaphor	8 (0.09)	38 (0.45)	50 (0.66)	6 (0.08)	57 (0.72)	11 (0.13)	1 (0.01)	12 (0.22)

## Discussion

The aim of this study was to determine and analyze the coaching behavior of dance teachers during various dance classes and rehearsals. An adapted version of the CAIS and additional focus of attention assessments were used to quantify frequencies of specific forms of instruction and feedback. Analyses of the determined coaching behaviors showed that the majority of dance teachers more often gave positive than negative feedback, that relatively few (open) questions were asked in relation to the amount of feedback provided, and that in general the elicited attentional focus was internal.

In the present study, teachers in all classes and rehearsals except one (i.e., Ballet 1), provided more positive compared to negative feedback. This finding contrasts with Klockare et al. (2011), who found that teachers in their sample, coming from comparable dance-styles as the current study, more commonly tended to provide more criticism and corrections than praising remarks. The positive-negative feedback ratios in the current study shows that these ratios also strongly differed between different classes/rehearsals. For both Ballet classes and Rehearsals, the positive-negative feedback ratio turned out to be almost 1 to 1 and numbers of negative feedback given were not negligible. These findings are in agreement with results of Van Rossum (2004) and Rafferty and Wyon (2006), demonstrating that there is still room for improvement for dance teachers to provide dance students with more positive feedback, or less negative feedback. On the other hand, improvisation had a positive-negative feedback ratio of 29 to 1. This could possibly be due to the didactical skills of the teacher. Another reason could be the characteristics of an Improvisation class (Carter, 2000). The freedom and responsibility afforded to a dancer during improvisation could reduce the possibilities for an “incorrect movement,” reducing the need for negative feedback. On the other hand, all other dance classes/rehearsals have specific dance-techniques. This means that within each technique one strives for the perfect execution of each movement. The traditional teaching method in these dance styles tends to focus on identifying imperfections and instructing the student on what he or she could do better. As Otte et al. (2020) argue, positive feedback should be prioritized and negative feedback moderated. The positive feedback, in relation to negative feedback could lead, among others, to more effective learning and better performance (Badami et al., 2012; Abbas and North, 2018).

During all but one (i.e., Improvisation) of the classes/rehearsals, more direct feedback than questions were used. These findings tie in with the characteristics of a traditional dance technique class (Meenan, 2013). However, it has been suggested that high feedback frequency can lead to dependence on the guidance provided by this feedback and hinder the development of an athlete’s self-regulation (Janelle et al., 1997; Otte et al., 2020). The use of questions, especially in the open form, could counteract these effects (Williams and Hodges, 2005; Neenan, 2009; Elferink-Gemser and Hettinga, 2017; Otte et al., 2020). However, the majority of questions asked during the analyzed classes and rehearsals were closed questions, except for the improvisation class, which had by far the highest number of open questions asked. The improvisation class consisted, in addition to a performing part, of a more theoretical part related to the dance exercises in the class. On the basis of open questions prior to an exercise, students were stimulated to think critically about what they do. Subsequently, open questions were also asked after the exercises. Questions such as “How does that make you feel?” and “What happened?” could stimulate the students to be actively involved in improving their dance improvisation. For the other classes and rehearsals, there seems to be room for change with regard to the high numbers of directly provided feedback and low numbers of (open) questions asked.

Both with instructions and feedback teachers can invite an external focus of attention, an internal focus, or use metaphors to steer focus of attention. Metaphors can potentially lead to an external focus of attention in dancers (Otte et al., 2020). Within the current study, not all comments made by the dance teachers could be classified in one of these categories. Although it appears from the literature that an external focus, which may include metaphors, leads to a more effective and efficient way of learning and performing (Wulf, 2013; Wulf and Lewthwaite, 2016), current data showed that the majority of teachers have a tendency to provide students with feedback stimulating an internal focus. These findings are in line with the results of Guss-West and Wulf (2016). Both absolute numbers and number of times per minute were highest for internal focus comments for six of the eight classes and rehearsals. Graham had the highest number of comments eliciting an internal focus of attention. The focus on the body in this dance style could explain the high number of internal focus comments, such as: “Rotate the arms out.” The improvisation class on the other hand, had the highest number of comments inviting an external focus of attention. The use of space was a central concept to this class. An example of an external comment was: “Face the barre in parallel.” The Jazz class had the highest number of metaphors used. An example of a metaphor was: “Now fly.” According to Wulf and Lewthwaite (2016), coaching behavior based on an external focus and metaphors, such as used in the Improvisation and Jazz classes, could have positive results for the performance level of dancers.

### Strength and limitations

This is the first study to determine the actual coaching behavior of dance teachers with the use of video and audio recordings, instead of using retrospective interviews and questionnaires as done in other studies. A strength of the current study is that coaching behavior was observed during actual real-life dance classes/rehearsals at a renowned contemporary dance university and that behavior was rated using the adapted CAIS, of which the interrater reliability was found to be good to acceptable. This means that a crude impression is provided on how dance classes are taught, although specific differences will always exist among teachers of similar classes and among dance academies. The present study shows that even at the highest level of dance education, insights from previous studies about coaching behavior in dance leave room for improvement. However, several limitations also need to be acknowledged. First, the current study was performed at one contemporary dance university, with six dance teachers. Although generalizability is partly limited, we have no reason to believe that it is very different at other dance universities. Based on the literature in the field of sports and motor learning, there are opportunities for change and improvement. Our study aimed to identify these opportunities for change and improvement. Nevertheless, it would be interesting to investigate whether numbers and types of instruction and feedback found in the current study between Ballet teachers and other dance styles are similar at other dance university. Second, the duration of each behavior was not determined. For example, setting an exercise was often counted as one instruction and could last for more than a minute. On the other hand, a simple comment as “Take your shoulder and rotate it,” lasting less than 2 s, was also counted as one instruction. Clear rules and instructions on how to use the modified CAIS made clear when to count one or multiple behaviors. Third, assessing the behaviors of dance teachers is complex. Comments could sometimes be classified in multiple categories. A simple “And” could be a *management* directed to the pianist to start playing, but could also be a *cue* to the students for a movement on the beat of the music. Clear descriptions of all categories and rules were essential and clarified how to assess each comment. Fourth, when interpreting the results, one should recognize that outcomes can be influenced by both the type of class or the didactical skills of the teacher. For example, behaviors observed during Improvisation may be influenced by the fact that this style does not have a strict technique in the way other classes and rehearsals have, and therefore may be presented in a different manner compared to the other classes. The type of class may, thus, be important factor determining teachers’ instructions and feedback. Regarding the didactical skills of the teacher in the present study, it is important to recognize that all teachers had extensive teaching experience, meaning more than 10 years, in the field of (pre)professional dance.

Fifth, there is a possibility that behaviors were influenced by the fact that teachers knew they were being filmed. Teachers were instructed to teach as they always do. However, videos used for the current study were part of a longer 4 weeks filming period. It may therefore be that teachers were already accustomed being filmed and referred to their traditional ways of teaching. Lastly, the current study did not look at any levels of dance performance and self-regulation of dance students. Measuring the performance level and self-regulation of a dancer is challenging. Artistry for example, is highly subjective and is considered to be an important factor in dance. For future research it should be established how the performance level and self-regulation of dancers could be measured.

### Practical implications

Assessing dance teachers’ coaching behavior is a time consuming but valuable activity. It provides actual measured verbal and non-verbal instructions and feedback, instead of using questionnaires about what dance teachers think they are saying and doing. Using such objective observations may inform teachers on how they could optimize their use of instructions and feedback, drawing upon performance psychology and skill acquisition research. As dance students often rely on the instructions and feedback of their dance teachers, a better self-regulation can lead to more independent dance students reaching a higher level more quickly in less training time. However, further research is needed to investigate whether such changes indeed have the anticipated positive effect on the self-regulation and performance of dance students. Furthermore, as the modified CAIS and focus of attention part were found to be reliable tools to determine dance teachers’ coaching behavior, it would be interesting to observe different levels of skilled dance teachers to examine if skill level determines the type of instructions and feedback utilized.

## Conclusion

This was the first time actual coaching behavior of dance teachers was collected and analyzed based on video and audio recordings. The current study shows that the coaching behavior of dance teachers can be determined and highlight the need to further investigate whether findings from sport and human movement sciences on coaching behavior are applicable to the dance context. Positive feedback was more often given than negative feedback; however, there is room for improvement. Moreover, the majority of dance teachers mainly elicited an internal focus of attention and asked closed questions or gave direct feedback. Rather, teachers should aim to provide dance students with a higher positive-negative feedback ratio and more external focus of attention comments and open questions. The use of such forms of coaching behavior could have a positive effect on the quality of dance trainings and therefore likely improve performance levels and self-regulation of dance students. To determine whether this is actually the case, follow up research is needed. The current study may provide a small first step in helping to understand how dance teachers in the future can optimize their practice designs.

## Data availability statement

The raw data supporting the conclusions of this article will be made available by the authors, without undue reservation.

## Ethics statement

The studies involving human participants were reviewed and approved by the Scientific and Ethical Review Board (VCWE) at the Vrije Universiteit Amsterdam. The patients/participants provided their written informed consent to participate in this study.

## Author contributions

BS: author master thesis in English (Breederode Hogeschool), data collection, video analyses and other analyses, and lead in converting thesis into manuscript for publication. LP: initial set up for research, author bachelor thesis (Human Movement Sciences, Vrije Universiteit Amsterdam), and written in Dutch and video analyses. JK: guidance and feedback throughout the bachelor and master thesis process and converting thesis into manuscript for publication. JS and RR: guidance and feedback throughout the master thesis process and converting thesis into manuscript for publication. RO: guidance and feedback throughout the bachelor and master thesis process and converting thesis into manuscript for publication. All authors contributed to the article and approved the submitted version.
