# Supplementary material for: An analysis of teachers’ instructions and feedback at a contemporary dance university
Source: Front Psychol. 2023 Apr 27;14:1133737. doi: 10.3389/fpsyg.2023.1133737 (PMC10174436; doi:10.3389/fpsyg.2023.1133737)
Supplement: Supplementary file 1 [file Data_Sheet_1.pdf]

Appendix 1

Numbers for all behaviors of the modified CAIS per class and rehearsal

Table 5.
The total absolute numbers and numbers of number of times per minute for all behaviors of the modified CAIS, per class and rehearsal.

	Ballet 1	Ballet 2	Graham	Improvisation	Jazz	Laban	Rehearsal 1	Rehearsal 2
**Total**	900 (10.31)	909 (10.78)	2239 (29.76)	355 (4.92)	1035 (13.13)	1112 (12.96)	517 (6.45)	808 (14.56)
**Man.**	88 (1.01)	29 (0.34)	66 (0.88)	39 (0.54)	176 (2.23)	114 (1.33)	29 (0.36)	57 (1.03)
**Ins.**	155 (1.78)	173 (2.05)	490 (6.51)	99 (1.37)	250 (3.17)	143 (1.67)	80 (1.00)	209 (3.77)
**Cor.**	25 (0.29)	81 (0.96)	35 (0.47)	1 (0.01)	32 (0.41)	95 (1.11)	70 (0.87)	42 (0.76)
**Cue.**	406 (4.67)	267 (3.17)	1216 (16.16)	11 (0.15)	322 (4.08)	500 (5.83)	24 (0.30)	155 (2.79)
**Open.**	2 (0.02)	6 (0.07)	2 (0.03)	59 (0.82)	2 (0.03)	9 (0.10)	2 (0.02)	1 (0.02)
**Closed.**	16 (0.18)	37 (0.44)	92 (1.22)	38 (0.53)	30 (0.38)	37 (0.43)	78 (0.97)	88 (1.59)
**Gpf.**	8 (0.09)	43 (0.51)	80 (1.06)	37 (0.51)	99 (1.26)	62 (0.72)	65 (0.81)	23 (0.41)
**Gnf.**	2 (0.02)	7 (0.08)	2 (0.03)	0 (0)	2 (0.03)	1 (0.01)	4 (0.05)	5 (0.09)
**Spf.**	3 (0.03)	11 (0.13)	2 (0.03)	21 (0.29)	1 (0.01)	2 (0.02)	4 (0.05)	0 (0)
**Snf.**	14 (0.16)	39 (0.46)	6 (0.08)	2 (0.03)	4 (0.05)	7 (0.08)	33 (0.41)	16 (0.29)
**Pos.**	126 (1.45)	152 (1.80)	148 (1.97)	36 (0.50)	80 (1.01)	90 (1.05)	67 (0.84)	161 (2.90)
**Neg.**	18 (0.21)	40 (0.47)	3 (0.04)	1 (0.01)	5 (0.06)	9 (0.10)	21 (0.26)	2 (0.04)
**Pa.**	8 (0.09)	7 (0.08)	6 (0.08)	0 (0)	8 (0.10)	10 (0.12)	18 (0.22)	14 (0.25)
**Hum.**	20 (0.23)	10 (0.12)	27 (0.36)	5 (0.03)	8 (0.10)	9 (0.10)	16 (0.20)	15 (0.27)
**Ans.**	3 (0.03)	3 (0.04)	7 (0.09)	1 (0.01)	13 (0.16)	16 (0.19)	4 (0.05)	15 (0.27)
**Hus.**	2 (0.02)	4 (0.05)	57 (0.76)	8 (0.11)	3 (0.04)	8 (0.09)	2 (0.02)	5 (0.09)

Appendix 2

Numbers before, during and after an exercise for all behaviors of the modified CAIS per class and rehearsal

Table 6.

The absolute numbers and numbers of number of times per minute before, during and after an exercise for all behaviors of the modified CAIS, per class and rehearsal.

		Ballet 1	Ballet 2	Graham	Improvisation	Jazz	Laban	Rehearsal 1	Rehearsal 2
MAN.	*before*	**50** (1.90)	**15** (1.00)	**33** (3.22)	**30** (1.27)	**81** (3.57)	**47** (2.36)	**8** (1.02)	**33** (1.04)
	during	**28** (0.57)	**6** (0.13)	**27** (0.44)	**2** (0.06)	**87** (1.86)	**57** (1.06)	**1** (0.04)	**17** (1.48)
	after	**10** (0.88)	**8** (0.35)	**6** (1.64)	**7** (0.49)	**8** (0.86)	**10** (0.81)	**20** (0.42)	**7** (0.58)
INS.	*before*	**73** (2.78)	**48** (3.25)	**64** (6.24)	**28** (1.19)	**88** (3.87)	**76** (3.82)	**13** (1.65)	**150** (4.71)
	during	**74** (1.50)	**107** (2.28)	**422** (6.88)	**71** (2.07)	**148** (3.16)	**53** (0.99)	**0** (0)	**24** (2.08)
	after	**8** (0.70)	**18** (0.80)	**4** (1.09)	**0** (0)	**14** (1.50)	**14** (1.14)	**67** (1.41)	**35** (2.88)
COR.	*before*	**1** (0.04)	**0** (0)	**0** (0)	**0** (0)	**2** (0.09)	**5** (0.25)	**0** (0)	**4** (0.13)
	during	**7** (0.14)	**43** (0.91)	**26** (0.42)	**1** (0.03)	**13** (0.28)	**65** (1.21)	**3** (0.12)	**9** (0.78)
	after	**17** (1.50)	**38** (1.68)	**9** (2.45)	**0** (0)	**17** (1.82)	**25** (2.03)	**67** (1.41)	**29** (2.39)
CUE.	*before*	**16** (0.61)	**12** (0.81)	**0** (0)	**0** (0)	**0** (0)	**2** (0.10)	**0** (0)	**18** (0.57)
	during	**390** (7.93)	**255** (5.43)	**1216** (19.83)	**11** (0.32)	**322** (6.88)	**498** (9.30)	**6** (0.24)	**136** (11.81)
	after	**0** (0)	**0** (0)	**0** (0)	**0** (0)	**0** (0)	**0** (0)	**18** (0.38)	**1** (0.08)
OPEN.	*before*	**1** (0.04)	**2** (0.14)	**2** (0.20)	**33** (1.40)	**1** (0.04)	**1** (0.05)	**1** (0.13)	**1** (0.03)
	during	**1** (0.02)	**1** (0.02)	**0** (0)	**11** (0.32)	**0** (0)	**2** (0.04)	**0** (0)	**0** (0)
	after	**0** (0)	**3** (0.13)	**0** (0)	**15** (1.06)	**1** (0.11)	**6** (0.49)	**1** (0.02)	**0** (0)
Closed.	*before*	**9** (0.34)	**20** (1.36)	**74** (7.22)	**17** (0.72)	**22** (0.97)	**20** (1.01)	**12** (1.53)	**54** (1.70)
	during	**1** (0.02)	**2** (0.04)	**6** (0.10)	**8** (0.23)	**2** (0.04)	**3** (0.06)	**0** (0)	**1** (0.09)
	after	**6** (0.53)	**15** (0.66)	**12** (3.27)	**13** (0.92)	**6** (0.64)	**14** (1.14)	**66** (1.39)	**33** (2.72)
GPF.	*before*	**1** (0.04)	**2** (0.14)	**0** (0)	**16** (0.68)	**9** (0.40)	**6** (0.30)	**0** (0)	**11** (0.35)
	during	**0** (0)	**24** (0.51)	**70** (1.14)	**11** (0.32)	**72** (1.54)	**40** (0.75)	**13** (0.52)	**0** (0)
	after	**7** (0.62)	**17** (0.75)	**10** (2.73)	**10** (0.71)	**18** (1.93)	**16** (1.30)	**52** (1.10)	**12** (0.99)
GNF.	*before*	**2** (0.08)	**0** (0)	**0** (0)	**0** (0)	**0** (0)	**0** (0)	**0** (0)	**2** (0.06)
	during	**0** (0)	**3** (0.06)	**1** (0.02)	**0** (0)	**0** (0)	**0** (0)	**0** (0)	**3** (0.26)
	after	**0** (0)	**4** (0.18)	**1** (0.27)	**0** (0)	**2** (0.21)	**1** (0.08)	**4** (0.08)	**0** (0)

SPF.	*before*	**0** (0)	**0** (0)	**0** (0)	**11** (0.47)	**1** (0.04)	**0** (0)	**0** (0)	**0** (0)
	during	**0** (0)	**3** (0.06)	**2** (0.03)	**0** (0)	**0** (0)	**0** (0)	**0** (0)	**0** (0)
	after	**3** (0.26)	**8** (0.35)	**0** (0)	**10** (0.71)	**0** (0)	**2** (0.16)	**4** (0.08)	**0** (0)
SNF.	*before*	**0** (0)	**0** (0)	**0** (0)	**0** (0)	**2** (0.09)	**1** (0.05)	**0** (0)	**3** (0.09)
	during	**1** (0.02)	**12** (0.26)	**3** (0.05)	**2** (0.06)	**2** (0.04)	**1** (0.02)	**0** (0)	**5** (0.43)
	after	**13** (1.14)	**27** (1.20)	**3** (0.82)	**0** (0)	**0** (0)	**5** (0.41)	**33** (0.70)	**8** (0.66)
POS.	*before*	**48** (1.83)	**12** (0.81)	**22** (2.15)	**8** (0.34)	**50** (2.20)	**34** (1.71)	**1** (0.13)	**71** (2.23)
	during	**47** (0.96)	**99** (2.11)	**114** (1.86)	**28** (0.81)	**9** (0.19)	**25** (0.47)	**1** (0.04)	**32** (2.78)
	after	**31** (2.73)	**41** (1.82)	**12** (3.27)	**0** (0)	**21** (2.25)	**31** (2.51)	**65** (1.37)	**58** (4.78)
NEG.	*before*	**2** (0.08)	**3** (0.20)	**0** (0)	**0** (0)	**0** (0)	**0** (0)	**0** (0)	**0** (0)
	during	**0** (0)	**10** (0.21)	**0** (0)	**1** (0.03)	**0** (0)	**0** (0)	**0** (0)	**0** (0)
	after	**16** (1.40)	**27** (1.20)	**3** (0.82)	**0** (0)	**5** (0.54)	**9** (0.73)	**21** (0.44)	**2** (1.64)
PA.	*before*	**1** (0.04)	**0** (0)	**0** (0)	**0** (0)	**0** (0)	**0** (0)	**0** (0)	**13** (0.41)
	during	**6** (0.12)	**3** (0.06)	**4** (0.07)	**0** (0)	**8** (0.17)	**9** (0.17)	**0** (0)	**0** (0)
	after	**1** (0.09)	**4** (0.18)	**2** (0.55)	**0** (0)	**0** (0)	**1** (0.08)	**18** (0.38)	**1** (0.08)
HUM.	*before*	**10** (0.38)	**4** (0.27)	**8** (0.78)	**0** (0)	**4** (0.18)	**7** (0.35)	**6** (0.76)	**4** (0.13)
	during	**3** (0.06)	**2** (0.04)	**15** (0.24)	**0** (0)	**0** (0)	**1** (0.02)	**0** (0)	**1** (0.09)
	after	**7** (0.62)	**4** (0.18)	**4** (1.09)	**2** (0.14)	**4** (0.43)	**1** (0.08)	**10** (0.21)	**10** (0.82)
ANS.	*before*	**3** (0.11)	**0** (0)	**5** (0.49)	**1** (0.04)	**12** (0.53)	**13** (0.65)	**2** (0.25)	**10** (0.31)
	during	**0** (0)	**0** (0)	**0** (0)	**0** (0)	**1** (0.02)	**1** (0.02)	**0** (0)	**0** (0)
	after	**0** (0)	**3** (0.13)	**2** (0.55)	**0** (0)	**0** (0)	**2** (0.16)	**2** (0.04)	**5** (0.41)
HUS.	*before*	**0** (0)	**0** (0)	**0** (0)	**2** (0.08)	**0** (0)	**0** (0)	**0** (0)	**0** (0)
	during	**2** (0.04)	**4** (0.09)	**57** (0.93)	**6** (0.17)	**3** (0.06)	**7** (0.13)	**2** (0.08)	**0** (0)
	after	**0** (0)	**0** (0)	**0** (0)	**0** (0)	**0** (0)	**1** (0.08)	**0** (0)	**5** (0.41)
